# Severe intoxication caused by sodium-glucose cotransporter 2 inhibitor overdose: a case report

**DOI:** 10.1186/s40360-019-0381-z

**Published:** 2020-01-09

**Authors:** Miho Nakamura, Junya Nakade, Tadashi Toyama, Masaki Okajima, Takumi Taniguchi

**Affiliations:** 1 Intensive Care Unit, Kanazawa University Hospital, 13-1 Takara-machi, Kanazawa, Ishikawa 920-8641 Japan; 2 Department of Hospital Pharmacy, Kanazawa University Hospital, Kanazawa, Ishikawa Japan; 3 Department of Anesthesiology and Intensive Care Medicine, Graduate School of Medicine, Kanazawa University, Kanazawa, Ishikawa Japan

**Keywords:** Sodium glucose transporter 2 inhibitors, Intoxication, Overdose

## Abstract

**Background:**

Sodium-glucose cotransporter 2 (SGLT2) inhibitors inhibit SGLT2, which is expressed in the proximal renal tubule, and thus reduce blood glucose levels by enabling the urinary excretion of excess glucose. SGLT2 inhibitors have been reported to suppress the complications of diabetes and reduce overall mortality. However, little is known about the types of symptoms that may occur in response to an overdose of an SGLT2 inhibitor. Here, we describe a case of intoxication caused by an overdose of an SGLT2 inhibitor.

**Case presentation:**

An otherwise physically healthy adult woman ingested an overdose of ipragliflozin, an SGLT2 inhibitor, and a polypill of olmesartan medoxomil, and azelnidipine in a suicide attempt. Although her blood ipragliflozin concentration was very high (9516.3 ng/mL) upon hospital arrival, her initial blood glucose level was normal, and she did not exhibit symptoms such as hypoglycemia or polyuria. Moderate renal dysfunction associated with an estimated glomerular filtration rate of 42.3 mL/min/1.73 m^2^ was observed. Thirty-six hours after ingestion, her blood ipragliflozin concentration decreased to a level equivalent to that observed after a therapeutic dose and her renal function improved almost simultaneously. After improvement in her renal function, the osmotic diuretic effect of the drug progressed. Her blood glucose level declined slightly but was in the normal range due to glucose administration. During the clinical course, fatal hypoglycemia was not observed.

**Conclusions:**

Our case showed that an overdose of an SGLT2 inhibitor caused toxic effects on renal function, but severe hypoglycemia was not observed. Additional cases of intoxication from SGLT2 inhibitors alone would be helpful to clarify the mechanism of intoxication.

## Background

Sodium-glucose cotransporter 2 (SGLT2) inhibitors inhibit SGLT2, which is expressed in the proximal renal tubule, and reduce blood glucose levels by enabling the urinary excretion of excess glucose [1]. Since SGLT2 inhibitors inhibit glucose reabsorption by SGLT2, urine osmotic pressure increases due to increased urinary glucose excretion, causing osmotic diuresis, which affects urine output and body fluid volume [2]. Since these drugs do not directly affect glucose metabolism or insulin secretion in the body, SGLT2 inhibitor monotherapy does not cause severe hypoglycemia [3].

SGLT2 inhibitors are becoming a key drug in the treatment of diabetes by suppressing diabetes complications including cardiovascular disease and all-cause mortality [4, 5]. On the other hand, little is known about the symptoms and clinical course in response to an overdose of SGLT2 inhibitors. Here, we report our experience with a case of SGLT2 inhibitor (ipragliflozin) overdose, in an otherwise healthy woman.

## Case presentation

A 32-year-old woman arrived at the emergency department with complaints of abdominal pain, nausea, and vomiting. Eight hours before arrival, with suicidal intent, she ingested 1500 mg of ipragliflozin, 800 mg of olmesartan medoxomil, and 640 mg of azelnidipine [a polypill of an angiotensin II receptor blocker/calcium channel blocker (ARB/CCB)], which was her mother’s medicine. She was treated with sodium valproate and chlorpromazine hydrochloride for her delusional disorder. She had previously experienced severe hypoglycemia in the event of an overdose of her mother’s medicine including an SGLT2 inhibitor along with a dipeptidyl peptidase-4 inhibitor and a sulphonylurea.

On admission, her vital signs were as follows: body temperature, 36.4 °C; heart rate, 47 beats/min; blood pressure, 66/26 mmHg; respiratory rate, 18 breaths/min and SpO_2_, 99% on room air. Except for slight drowsiness and hypotension, her physical examination was normal. Blood biochemistry showed the following: glucose, 126 mg/dL; urea nitrogen, 7.1 mmol/L; creatinine, 108 μmol/L; estimated glomerular filtration rate 42.3 mL/min/1.73 m^2^; sodium, 137 mmol/L; potassium, 4.8 mmol/L; calcium, 2.3 mmol/L; alanine aminotransferase, 32 IU/L; aspartate aminotransferase, 33 IU/L and creatine kinase, 50 IU/L. Urinalysis showed the following: specific gravity, 1.029; protein, negative; glucose > 1000 mg/dL; ketone, 0 mg/dL; bilirubin, 0 mg/dL; nitrite, negative and leukocytes, negative. An arterial blood gas analysis showed the following: pH, 7.40; pCO_2_, 43.0 Torr; HCO_3_^−^, 26.1 mmol/L; base excess, 1.6 mmol/L and anion gap, 8 mmol/L. A transthoracic echocardiogram revealed normal cardiac function and an inferior vena cava diameter > 20 mm.

The patient was admitted to the psychiatric ward because of her suicidal intent. Although she was treated with norepinephrine at 0.25 μg/kg/min, she still had hypotension. She was transferred to our intensive care unit (ICU) 20 h after ingestion.

**Fig. 1:**
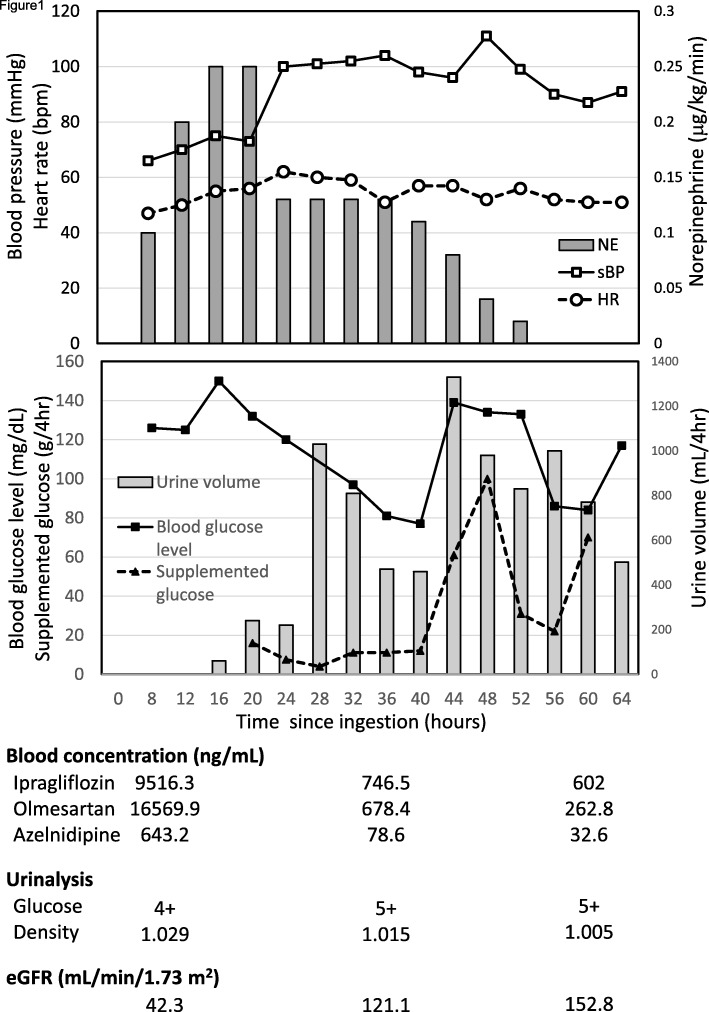
Clinical course and blood drug concentrations. HR: heart rate, NE: norepinephrine, sBP: systolic blood pressure. eGFR: estimated glomerular filtration rate, Urinalysis glucose 4+: over 1000 mg/dL, 5+: over 2000 mg/dL

After the treatment in the ICU, her blood pressure recovered, and we could discontinue norepinephrine as her blood concentration of ARB/CCB decreased 56 h after ingestion (Fig. 1). The patient’s blood concentration of ipragliflozin was highest at the time of admission (9516.3 ng/mL), but her blood glucose level was normal. Thirty-six hours after ingestion, the blood concentration of ipragliflozin had decreased to 746.5 ng/mL, equivalent to the level of a therapeutic dose, and her renal function improved simultaneously. After improvement of renal function, the osmotic diuretic effect of the drug progressed and her blood glucose level declined. Her blood glucose level was at the lower end of the normal range, but continued to decline after 40 h of ingestion, so she was treated with glucose prophylactically. When glucose was added to maintain her blood glucose level, a marked increase in urine volume was observed (Fig. 1). Crystalloid therapy was administered temporarily to treat the dehydration resulting from this diuretic effect. Unlike the previous admission due to overdose of SGLT2 inhibitors along with dipeptidyl peptidase-4 inhibitors and sulfonylureas, severe hypoglycemia was not observed during the clinical course. She was discharged from the ICU 64 h after ingestion and discharged from the hospital on day 17.

## Discussion and conclusion

We experienced a case of an overdose of ipragliflozin, an SGLT2 inhibitor, along with ARB/CCB. SGLT2 inhibitors are known to be a risk factor for hypoglycemia even with the therapeutic dose. However, in this case, a very high blood concentration of ipragliflozin on admission did not result in hypoglycemia during the clinical course. Temporary renal dysfunction and hypotension may have resulted in the attenuated glucose-lowering effect.

SGLT2 inhibitors have a glucose-lowing effect, but there is little risk of severe hypoglycemia due to SGLT1 reabsorption of glucose which occurs in the distal part of the proximal tubule [3]. One of the reasons the patient did not show severe hypoglycemia might be because she did not take other glucose-lowering agents at the same time which have been reported to induce severe hypoglycemia [6]. She previously experienced severe hypoglycemia in the event of an overdose of an SGLT2 inhibitor along with dipeptidyl peptidase-4 inhibitors and sulfonylureas.

The other mechanism that can explain the absence of hypoglycemia might be the renal dysfunction. SGLT2 inhibitors temporarily decrease renal function due to the contractile action of afferent arterioles [7]. In addition, the effect of ARB on decreasing the efferent arteriolar tone as well as the systemic hypotension due to ARB and CCB overdose may have reduced the glomerular filtration rate, which resulted in decreased urine glucose excretion [8, 9]. Her bradycardia was probably due to the overdose of azelnidipine, an L-type calcium channel blocker [10]. The patient did not experience severe hypoglycemia, which may be due in part to the reduced renal function and systemic hypotension from the concomitant intake of ARB/CCB; it was consistent with the subsequent mild hypoglycemia and osmotic diuresis requiring glucose supplementation after the recovery of renal dysfunction and hypotension. Another reason for avoiding hypoglycemia might be an overdose of CCB [9]. The increasing blood glucose due to azelnidipine overdose could mask the hypoglycemia due to the SGLT2 inhibitor.

SGLT2 inhibitors will be essential for diabetes treatment. The widespread use of this drug class has led to concerns regarding intoxication. In this case, a concomitant intake of other drugs affecting renal function made it difficult to clarify the pure effect of the SGLT2 inhibitors. To investigate the mechanism in depth, it is necessary to examine overdoses with SGLT2 inhibitors alone.

## Data Availability

The data are not publicly available due to restrictions as they contain information that could compromise the privacy of the patient.

## Abbreviations

ARB: A combination angiotensin II receptor blocker
CCB: Calcium channel blocker
ICU: Intensive care unit
SGLT2: Sodium-glucose cotransporter 2
